# Temperature and Microstructure Evolution in Gas Tungsten Arc Welding Wire Feed Additive Manufacturing of Ti-6Al-4V

**DOI:** 10.3390/ma12213534

**Published:** 2019-10-28

**Authors:** Corinne Charles Murgau, Andreas Lundbäck, Pia Åkerfeldt, Robert Pederson

**Affiliations:** 1 Department of Engineering Science, University West, 46129 Trollhättan, Sweden; corinne.charlesmurgau@gmail.com (C.C.M.); robert.pederson@hv.se (R.P.); 2 Division of Mechanics of Solid Materials, Luleå University of Technology, 97181 Luleå, Sweden; 3 Division of Materials Science, Luleå University of Technology, 97181 Luleå, Sweden; pia.akerfeldt@ltu.se; 4 GKN Aerospace Engine Systems, 46181 Trollhättan, Sweden

**Keywords:** additive manufacturing, titanium, Ti-6Al-4V, microstructural modeling, metal deposition, finite element method

## Abstract

In the present study, the gas tungsten arc welding wire feed additive manufacturing process is simulated and its final microstructure predicted by microstructural modelling, which is validated by microstructural characterization. The Finite Element Method is used to solve the temperature field and microstructural evolution during a gas tungsten arc welding wire feed additive manufacturing process. The microstructure of titanium alloy Ti-6Al-4V is computed based on the temperature evolution in a density-based approach and coupled to a model that predicts the thickness of the α lath morphology. The work presented herein includes the first coupling of the process simulation and microstructural modelling, which have been studied separately in previous work by the authors. In addition, the results from simulations are presented and validated with qualitative and quantitative microstructural analyses. The coupling of the process simulation and microstructural modeling indicate promising results, since the microstructural analysis shows good agreement with the predicted alpha lath size.

## 1. Introduction

The Gas Tungsten Arc Welding (GTAW) wire feed additive manufacturing (AM) process involves the deposition of metal material using a tungsten arc as an energy source. GTAW belongs to the group of Direct Energy Deposit (DED) methods in the family of AM processes. A solid metal wire is fed through a conventional wire feeder and deposited layer-by-layer onto a substrate. The energy source is electrical heating, which melts the metal wire as well as the base material. The addition of multiple layers produces a dense near-net-shape part. GTAW wire feed additive manufacturing has a high deposition rate [1]. Flexibility and cost savings in manufacturing are important driving forces in the development of such techniques [2].

The titanium alloy, Ti-6Al-4V, is extensively used in aerospace applications because of its high strength to weight ratio [3]. With an appropriate protective environment to minimize the oxygen content, this alloy shows good weldability and has also been shown to be suitable for additive manufacturing. 

In additive manufacturing, multiple layers are melted on top of each other in a layer-by-layer manner, inducing a number of heating-up/cooling-down cycles in the manufactured material. Since Ti-6Al-4V is a two-phase alloy consisting of 90–95% of hcp-α phase and 5–10% bcc-β phase at room temperature, the final microstructure of the material after being additive manufactured can exhibit a variety of microstructures, depending on the conditions of the thermal cycles. For the industry to fully adopt additive manufacturing, and to be able to qualify titanium parts for aerospace applications, a complete understanding of the mechanical behavior and control of the resulting properties are prerequisites.

Process simulations provide information on how to manufacture components in order to achieve the required properties and support the development and understanding of the manufacturing process itself. Finite Element (FE) simulations, a conventional method used in the modeling of welding processes, particularly for larger components, are applied on the macro scale [4,5,6,7]. In the present work, a validated FE model presented by Lundbäck and Lindgren [4] is used to predict the thermal field that, in turn, determines the microstructural evolution. A number of different strategies exist regarding how to include detailed and explicit modeling on a microscopic scale in a macroscopic simulation. One such strategy is to use submeshes located at the nodes of the FE model [8], while another is to use dual-mesh methods that place microstructural domains at the nodes of a macroscopic FE calculation [9,10]. However, with these strategies, the calculations become very cumbersome if a large component is to be simulated. Therefore, for industrial needs, it is more pragmatic to use a density type of model, also called the internal state variable approach by Grong and Shercliff [11]. In this work, a density-type approach is used in order to model the microstructure on a larger scale. Moreover, it facilitates the future combination of the microstructural model with a mechanical model to compute material properties.

The aim of the present study has been to, for the first time, couple the process simulation model, developed by the authors [4], with a microstructural model, which has also been developed by the authors [12], to predict the alpha lath size in the GTAW wire feed AM process. The results were validated by building a well-defined, 10-layer-high weld sequence, in which, at specific positions, its microstructural features have been characterized and compared to the predicted results.

## 2. Process Description and Microstructural Characterization

### 2.1. Experimental Setup

**Figure 1:**
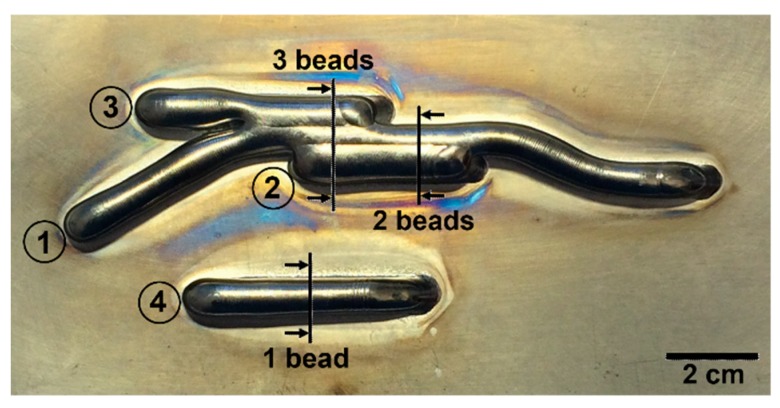
The built feature consisting of the four sequences of ten layers in height, including the starting positions and sequence order. The arrows indicate the position for microstructural characterization of each cross-section.

The Ti-6Al-4V was wire-feed deposited on a 3.25 mm-thick plate using a tungsten arc weld heat source. Four weld sequences with a height of 10 layers were continuously added onto the plate, as presented in Figure 1. Each layer is approximately 0.7 mm in height. Continuous addition means that no waiting time between each deposited layer was applied, except for the short time during welding torch movement to the new starting position. In order to avoid oxidation and alpha case formation during the building process, the oxygen level was kept below 10 ppm in the building chamber. This was achieved by applying an over-pressurized argon gas flowing through the chamber. The weld passes are numbered 1 to 4; Figure 1 indicates each starting point. The building sequence is such that walls 1 through 4 are deposited sequentially for the current layer and the structure is built in a layer-by-layer fashion. In total, ten layers were deposited for each wall.

### 2.2. Microstructural Characterization

The microstructures of three selected cross-sections, as indicated in Figure 1, were characterized. The location of each cross-section was selected so that the microstructure of different widths (one, two, and three weld beads) of the built material could be characterized. Each sample was mounted, ground, and polished using conventional methods for titanium alloys, and finally, etched with Kroll’s reagent (1 mL HF, 2 mL HNO_3_, and 100 mL distilled H_2_O) to reveal the microstructure.

The microstructural characterization was carried out in a light optical microscope (NIKON eclipse MA200) equipped with image analysis software (NIS Elements Basic Research). Large-area mapping was first carried out to capture overview images of the cross-sections. Thereafter, the microstructural characterization was planned in detail and the areas to be analyzed were decided. In each area, ten images at 1000-times magnification were captured and, in each image, the α lath thickness was measured at 25 randomly selected sites, yielding 250 α lath measurements in total. In addition, the fraction of grain boundary α was assessed for the single weld bead cross-section through manual measurements. The working sequence was as follows: first, large-area mapping (approximately 110 images) of the selected region at 500-times magnification was carried out at the highest resolution (2560 × 1920), and then the grain boundary α was carefully marked and colored red using an image editing software (Adobe Photoshop CC 2015) and digital zoom. The fraction of grain boundary α was subsequently calculated by comparing the total number of pixels to the number of red pixels in the images.

## 3. Process Modeling

The similarities between the GTAW process and multipass welding permit the application of welding simulation techniques. Computational Welding Mechanics (CWM) establishes methods and models that are applicable for the control of welding processes to obtain optimal mechanical performance. The book by Lindgren [13] describes different modeling options and strategies.

### 3.1. Thermal Model

For the thermal model, the weld pool details are replaced by a heat input model. The modeling is thus considerably simplified, but is still able to create a model fit for the purpose. The implemented logic and model is thoroughly described in [4]. Some noteworthy clarifications to the process model are briefly mentioned below.

The heat input model is the commonly-used double-ellipsoid with Gaussian distribution, as proposed by Goldak et al. [14]. An adaptive rescaling of the heat input through the efficiency factor is, when needed, used to control the variation of the heat input function due to the rather coarse mesh used for the discretization of the model. The parameters for the heat input and heat dissipation are the same as presented in [4]. For convenience, they are listed in Table 1, where *Q* is the nominal effect of the heat source, *η* is the efficiency factor, *h* is the heat transfer coefficient, and *e* is the emissivity. The *a, b, c_f_*, and *c_r_* parameters define the geometry of the double ellipsoid. The model was validated thermally in [4], and found to be in good agreement with the measurements.

**Figure 2:**
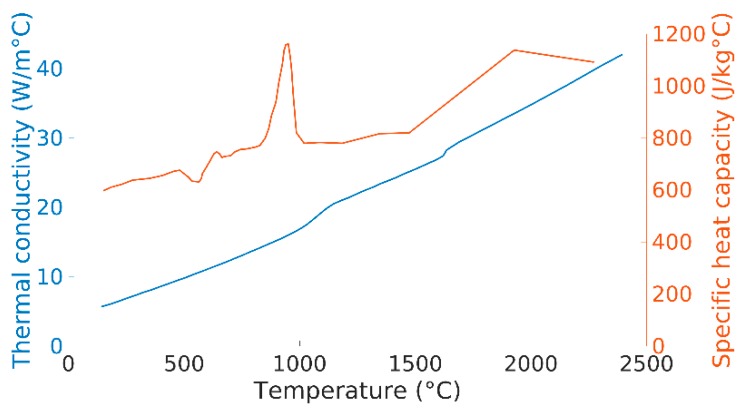
Material data used in the thermal model.

The material data that has been used in the thermal model is from [15]. The thermal conductivity and the specific heat capacity can be seen in Figure 2. Whenever the temperature in the model is outside the given data, a cut-off value is used, i.e., the last known value. A latent heat of 290 J/kg is applied in the transition between the solidus and liquidus temperatures, which are set to 1604 °C and 1700 °C, respectively. 

The number of nodes and elements in this model are approximately 19,000 and 13,000, respectively. The element type is 8-noded, fully integrated, hexahedral elements. The FE-software (version, company, city, country) used in this work, MSC.Marc (version, company, city, country), and its pre- and post-processor Mentat, have a number of interfaces for user-defined subroutines. The heat input is defined via user subroutines. The microstructural model that will be described in the next section is also defined via these user subroutines.

### 3.2. Microstructure Model

The phase evolution is computed during heating, cooling, and during repeated re-heating and cooling. During heating, when the temperature exceeds about 700 °C, the α phase starts to transform to the β phase [16]. Normally, during AM as well as welding, the heating rates are too fast for an equilibrium to exist. This means that Xβ < Xβ-eq, even at temperatures exceeding the so-called β-transus of the alloy. Above the β-transus temperature at equilibrium, only the β phase exists. During cooling, existing β phase transforms into α phase. For slow to moderate cooling rates, the transformation is diffusion controlled. The initial α phase normally nucleates at the prior β grain boundaries and continues to grow along these grain boundaries before starting to grow into the prior β grains, in a lamellar morphology. This lamellar type of microstructure is called the Widmanstätten microstructure here. For very high cooling rates, non-diffusional transformation of β → α phase takes place; this type of α phase is, therefore, defined as martensitic α, i.e., the microstructure will be martensitic. For moderate to high cooling rates, a massive transformation of β → α can occur [17]. This transformation is not included in the model. Solid–solid phase changes, on heating as well as on cooling, are mostly characterized by transformation mechanisms of nucleation and growth processes. The β phase decomposition to α Widmanstätten and α at prior β grain boundaries, as well as the β formation during heating, are all diffusion-controlled processes [18]. In contrast, α martensite formation from the β phase is a diffusionless transformation.

#### 3.2.1. Solid–Solid Phase Change Model

The diffusional phase transformations of β to α and α to β are evaluated using a modified Johnson–Mehl–Avrami theory. This equation is strictly valid only under isothermal transformation; therefore, the additivity principle has been adopted, and discretization into a series of smaller isothermal steps is used during temperature variations. The rule of additivity [16] is commonly used to calculate non-isothermal transformation from isothermal transformation data using simple rate laws. It should be noted that the additivity rule should be applied only under certain conditions in which the reaction is additive [10,16,18]. Grong and Shercliff [11] compared the additivity approach to numerical solutions when modeling microstructural state variables, with a focus on applications in heat treatments and welding. Even though discrepancies are observed, the approximation is evaluated to be sufficient for many problems, particularly when the constant data is estimated against experimental data for microstructure. The generalization steps of the Johnson–Mehl–Avrami equation used in this work are thoroughly detailed in [12]. The incomplete transformations toward equilibrium at the current temperature are circumvented by normalizing the equations. The interaction between simultaneous transformations is handled by assuming that the current fraction of the resultant phase is taken relative to the total content of the transforming phase.

The diffusionless martensite formation was chosen to be modeled by the classical Koistinen–Marburger equation, for which a direct incremental formulation of the equation, shown to be simpler and equally well accurate [19], was chosen. The overall equations for the model and their discretization are detailed in [12].

#### 3.2.2. Morphology Parameter: Alpha Lath Thickness

The morphology size parameter associated with Widmanstätten α phase, i.e., the α lath thickness parameter, was modeled by a simplified energy model approach [20]. The α phase formation temperature is here, as a first approximation, considered dominant in determining the α lath thickness. The empirical Arrhenius equation is used to express the temperature dependence of the α lath thickness. A first approximation for kinetic parameters was used in [20]; the proposed values seemed to be outside of the expected parameters’ dimensions. Irwin et al. [21] updated the values for the parameters after additional optimization supported by a new set of experimental results. The new suggested values, Arrhenius prefactor k = 1.42 µm and activation temperature R = 294 K, appear to have appropriate dimensions, and are thus used in this work. The equation and its explicit form used in the model can be found in [20].

#### 3.2.3. Implementation Strategy

**Figure 3:**
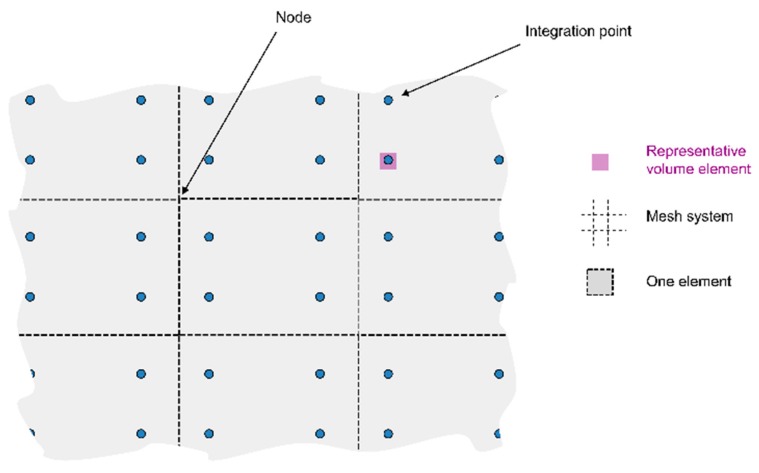
Schematic illustration of density type of model.

The development of the microstructural model is based on the finite element method, and is supported by the features in the software MSC.Marc. The microstructure is homogeneously described by state variables associated at each of the integration points of the finite element mesh. This approach means that Representative Volume Elements (RVE) (see Figure 3) are considered at each integration point of the elements. The calculated value corresponds to an average behavior over this domain. For example, the phase fraction in an integration point then corresponds to the fraction of the phase in the RVE connected to this particular integration point.

Four state variables are used in the model to represent the fraction of microstructural constituents. One more state variable, denoted as the size parameter, is used for the α lath thickness. The state variable denominations and the size parameter can be seen in Table 2. The α phase can form a number of different types of microstructure, but for modeling purposes, a choice to approximate the diffusional-formed α phase into a twofold-different microstructure is made, namely (i) grain boundary α (α_gb_), and (ii) Widmanstätten (α_w_) microstructure. The grain boundary α (α_gb_) is the α phase that is formed in the prior β grain boundaries. The Widmanstätten (α_w_) structure represents the α phase that forms inside the prior β grains (which is here considered to include both colony type and basket weave microstructures). The α martensite (α_m_) structure and the β phase are also represented.

**Figure 4:**
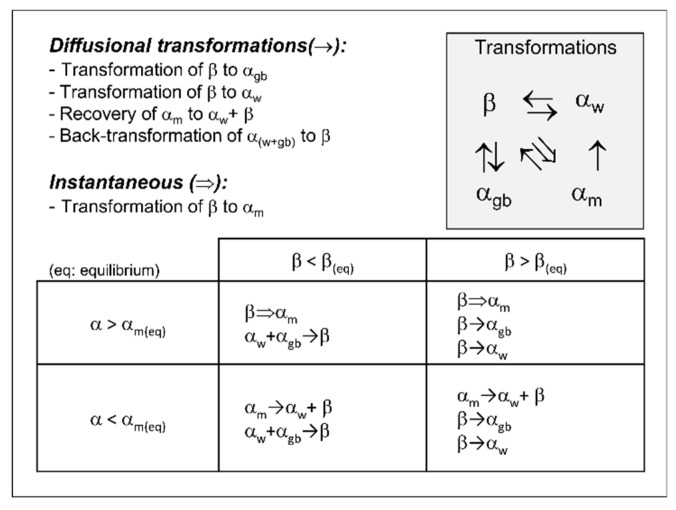
Transformation process and constituent interactions.

The interactions between the constituents are schematically presented in the upper-right square of Figure 4. In this work, four different diffusional transformations and one non-diffusional transformation are implemented. The transformation processes and microstructural constituent interactions that are implemented in the model are shown in the table in Figure 4. A detailed description of the logic and the overall model can be found in [12].

### 3.3. Adaptive Time Sub-stepping

To optimize the solution routine and reduce the computational time, an adaptive sub-stepping to calculate the microstructural model was adopted. To improve the accuracy, a refined time stepping is used when the temperature is in the phase transformation region. When necessary, each thermal step is thus divided into several smaller thermal sub-steps, assuming a linear temperature variation inside the original time step. 

## 4. Results and Discussion

### 4.1. Microstructural Analysis

**Figure 5:**
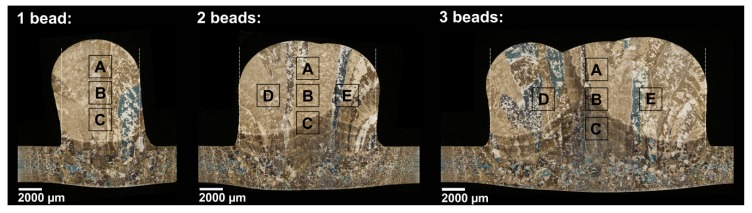
Light optical micrographs showing the characterized cross-sections indicated in Figure 1. The different areas, denoted as A, B, C, D, and E, correspond to the location of the measurements presented in Table 3.

**Figure 6:**
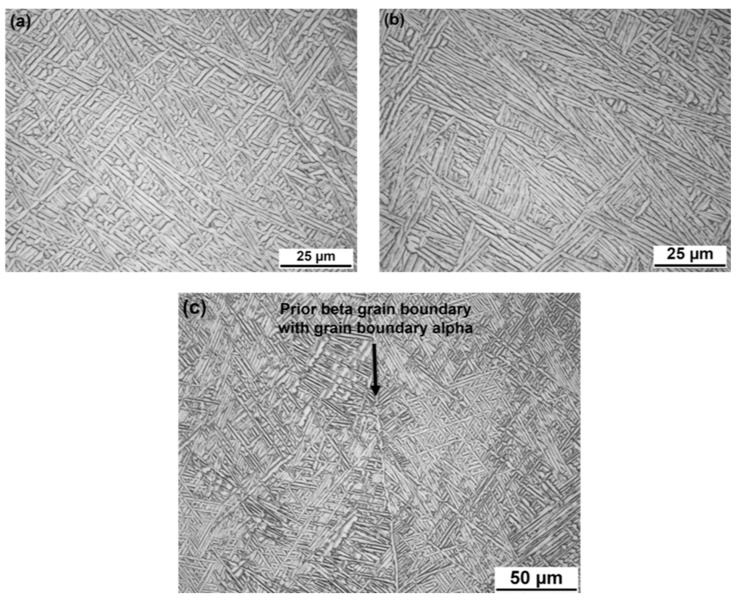
Light optical micrographs showing the microstructure of the GTAW wire feed built Ti-6Al-4V consists of fine α laths in the form of (**a**) basketweave α or (**b**) colony α structure. The prior β grain boundary is decorated with grain boundary α (**c**).

The microstructural analysis was performed on the areas highlighted in Figure 5, denoted A, B, C, D, and E. In general, the microstructure of the GTAW wire feed built Ti-6Al-4V consists of large, columnar, prior β grains that grow in the temperature gradient direction, through several layers. The directions of the columnar prior β grains can be seen in Figure 5. The prior β grains, seen as large areas of different color and/or contrast in Figure 5, are increasingly oriented towards the sides of the cross-section because of the temperature gradient. Within the prior β grain, fine α laths are observed in the form of either basketweave α structure (see Figure 6a) or colony α structure (see Figure 6b). However, only small regions of colony α are observed for the GTAW wire feed built Ti-6Al-4V, with the main part of the α laths being in the form of the basketweave α structure. The prior β grain boundaries are decorated by grain boundary α, as shown in Figure 6c. It is noteworthy that the thickness and prevalence of grain boundary α varies in different cross-sections. In some regions, the grain boundary α is continuous, as it is in Figure 6c, while it is absent or discontinuous in other grain boundaries.

The result of the quantitative microstructural characterization is summarized in Table 3. In general, only a small difference is observed when comparing the different areas. The variation is within the range of the standard deviation. However, one tendency is that slightly thicker α laths form with increasing thickness of deposited material (number of beads). This could be explained by the increased number of heating cycles due to the additional beads that allow the diffusional growth of the α laths to continue for a longer time. The α lath measurements are consistent with those previously reported [22] (0.8 ± 0.2 mm) for GTAW wire feed built Ti-6Al-4V, not for the same, but for similar process conditions. In contrast to the α lath thickness, a large variation of the grain boundary α fraction is observed. The fraction of grain boundary α depends on the location of the prior β grains and, furthermore, on the size of the prior β grains. As seen in Figure 5, the widths of the prior β grains vary significantly within the cross-sections, making the fraction estimation highly sensitive to the location of the evaluated area. Moreover, because of its limited thickness, the grain boundary α could be difficult to discern, which may have been the case for some limited regions of the areas investigated in the present study. For future work, it is therefore recommended that the validation be carried out of the grain boundary α on a deposited material with a slower cooling rate, for which a thicker grain boundary α is expected to be better defined, and thus, easier to measure.

### 4.2. Microstructural Simulation

**Figure 7:**
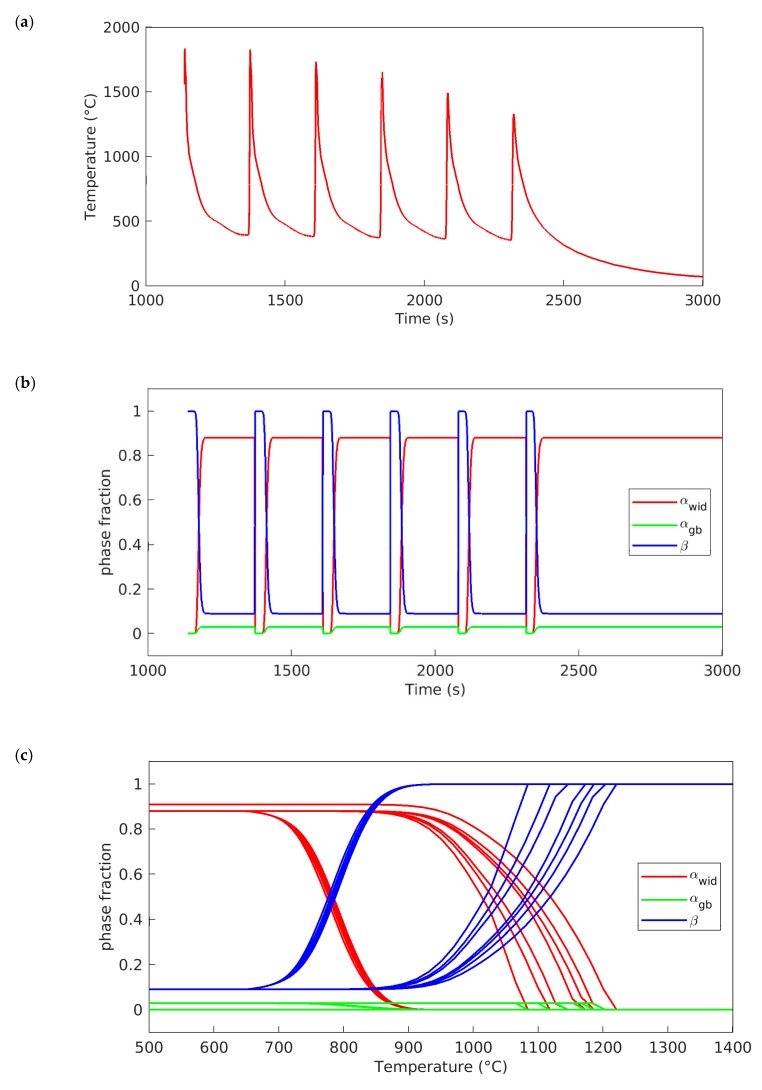
Simulation results at a node positioned in one weld bead cross-section positioned in the B area (Figure 5). (**a**) Temperature variations vs time. (**b**) Corresponding simulated α_gb_, α_wid_, and β phase fractions vs time. (**c**) Corresponding simulated α_gb_, α_wid_, and β phase fractions vs. temperature.

The additive manufacturing process is characterized by cyclic temperature variations, leading to repetitive phase transformations and microstructural changes in the deposited material and substrate. Temperature history is thus the main factor when modeling the microstructure. Continuous microstructural modeling allows for the microstructural changes that occur during processing to be followed. While microstructural analysis provides information about the results after the additive manufacturing process, the microstructural model, by following the entire deposition process, gives continuous information about the microstructure changes during the additive manufacturing process. The temperature history experienced at a selected point during additive manufacturing (see Figure 7a) is representative of a typical temperature profile experienced by the deposited material. The location of this point is in area B in the single weld bead sample in Figure 5. In Figure 7b,c, the simulated phase transformations versus time and temperature, respectively, can be seen. As explained earlier, during cooling, the β phase transforms into a mixture of α_gb_ and α_wid_. The phase fraction of each microconstituent depends on the cooling rate, i.e., a faster cooling rate promotes more α_wid_ than α_gb_, and vice versa. During the following heating sequence, the α_gb_ and α_wid_ transform to the β phase, which cyclically transforms again to α_gb_ and α_wid_ over the temperature history. It is important for the numerics in the microstructural model that the temperature change during a time step is not too large. Therefore, an internal time sub-stepping logic was developed to control this. In the current work, a maximum temperature change within the phase transformation region of 5 °C is allowed. If the temperature change in the thermal solution exceeds this value, the time step will be subdivided internally. The authors have observed that, for this specific set-up, the model yields a slightly different result when using the sub-stepping logic during the heating cycle. However, during the cooling phase, they are nearly identical. For another type of AM process, e.g., powder bed, the sub-stepping logic is probably of more importance, as the cooling ratios are much higher.

**Figure 8:**
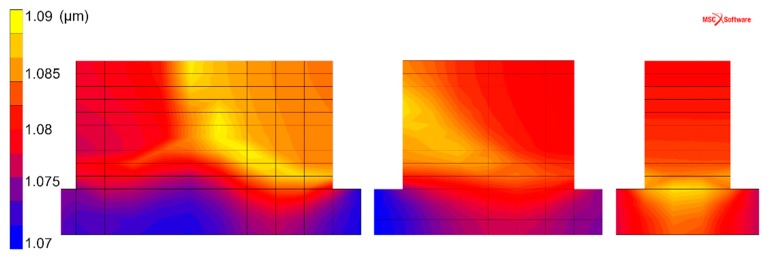
Simulated α lath thickness (µm), cross-sections with walls of widths of one, two, and three weld beads.

**Figure 9:**
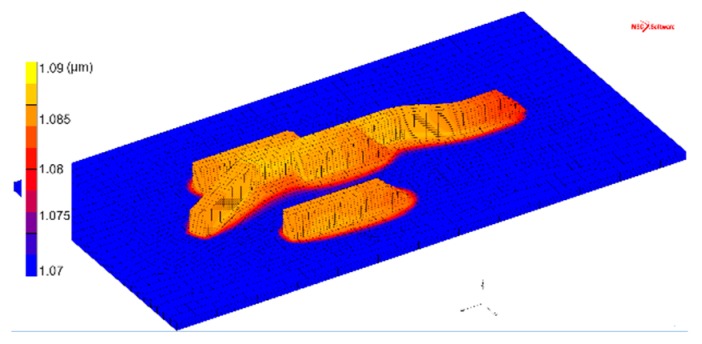
Simulated α lath thickness (µm) variation in the additive manufactured part.

Figure 8 and Figure 9 show the simulated results of the α lath thickness. Despite the simplicity of the chosen model, the simulation results agree well with the experimental measurements. The simulated thickness, evaluated to be approximately 1.1 µm, is in agreement with the 1.0 to 1.1 µm obtained by microstructural analysis. It is also interesting to note that the α lath thickness shows a tendency to increase with increasing wall width, i.e., when built with more weld beads. A similar trend is found in the experimental evaluation.

**Figure 10:**
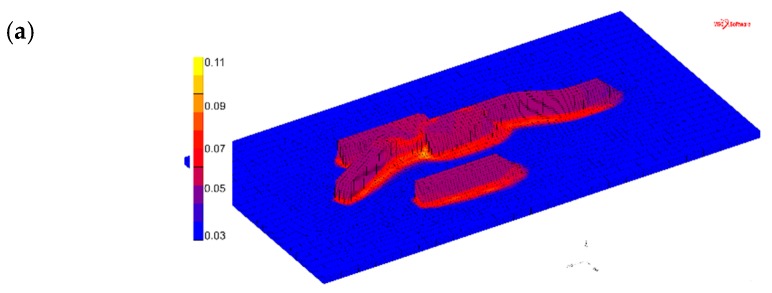

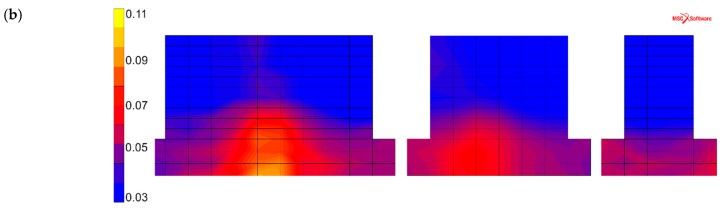
Simulated αgb phase fraction after wire feed additive manufacturing. (**a**) Complete view of the sample. (**b**) Cross-sections of one, two, and three weld-beads-wide walls.

The model predicts 3–6% α_gb_ in the deposited material, as seen in Figure 10, and the experimental measurements indicate α_gb_ fractions between 0.05–0.2%, as seen in Table 3. The small amount of α_gb_ phase and the large variation of the α_gb_ amount that was observed experimentally result in some uncertainty. However, it is obvious that the model overpredicts the amount of α_gb_. As already stated in the microstructural analysis section, a more accurate validation case could be achieved by using deposited material containing a thicker grain boundary α, which could be obtained in slowly-cooled deposited material.

**Figure 11:**
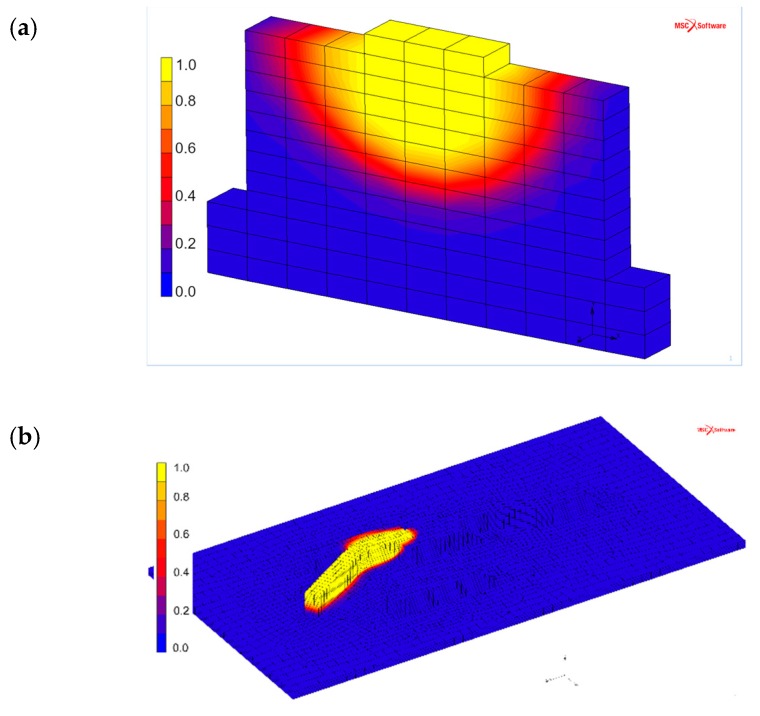
Simulated β phase fraction during wire feed additive manufacturing. (**a**) Cross-section of the three-weld beads wall. (**b**) Complete view of the sample.

An example of the results that can be obtained during the additive manufacturing process by using simulations is shown in Figure 11. The effect of the newly-deposited layer on the previously deposited layers is presented here. The simulated β phase fraction illustrates the ongoing phase transformations that are taking place while depositing the consecutive metal layers. The model, as well as the microstructure characterization, show no martensitic areas in the microstructure for the current manufacturing parameters.

The presented method for the modeling of wire feed additive manufacturing has been implemented into subroutines that can be evoked using commercial, general-purpose, finite element software. Moreover, microstructural modeling increases our understanding of the microstructural evolution, not only for post building, but also during the additive manufacturing process. The α lath thickness has been successfully predicted. The β phase transformation model (equivalent to the complementary total α phase transformation model) was validated in a previous publication [12] and used by others, e.g., [21,23]. However, the α_gb_ phase transformation part of the model is yet to be validated.

In addition, the microstructural model is an important tool that enables continued additive manufacturing process development to obtain improved material properties. Because physical and mechanical properties are dependent on the microstructure, it is also interesting to consider the microstructural development during the AM process. Then, the model can be coupled with a flow stress model needed in a thermo-mechanical analysis. The plastic properties will then depend on the current temperature and microstructure. The microstructural model is coupled with the dislocation density-based plasticity model presented in [24]. This type of constitutive model has a natural handshake with microstructural models; the coupling and application of these two models are presented in [25].

## 5. Conclusions

In this work, a process simulation model was coupled to a microstructural model. A density type of microstructural model was chosen in the current work. The main reason for this was that it should be possible to apply the model to industrial applications on a macroscopic level. The study presented herein shows promising results, since the size of the predicted alpha laths correlated very well with the microstructural analysis. Other types of models, such as sub-models or dual-mesh models, can give more detailed information about the microstructure, particularly the morphology; however, they typically become very computationally demanding, and are suitable for the whole field in an industrial application. In addition, the density type facilitates the combination of the microstructural model with a mechanical model to compute material properties, which is also shown in [25]. 

## Figures and Tables

**Table 1:** Parameters for the heat input and heat dissipation.

*Q* (W)	*η*	*a* (m)	*b* (m)	*c_f_* (m)	*c_r_* (m)	*h* (W/m^2^K)	*e*
896	0.58	0.004	0.0012	0.004	0.006	18	0.05

**Table 2:** Microstructural parameters and morphology description used in this work.

Phase Constituents		Type	State Variable	Size Parameters
**α**	**Diffusional α**	Grain boundary	X_αgb_	-
		Intergranular, Basket-weave, Colony	X_αw_	Lath thickness, t_α-lath_
	**Non-diffusional α**	Martensite	X_αm_	-
**β**		-	X_β_	-

**Table 3:** The result of the quantitative material characterization of the three cross-sections containing widths of one, two, and three beads, respectively.

Cross Section	Area	Average α Lath Thickness (μm)	Grain Boundary α Phase Fraction (%)
One bead	A	1.1 ± 0.4	0.21
	B	1.0 ± 0.3	0.11
	C	0.9 ± 0.3	0.05
	All	1.0 ± 0.3	-
Two beads	A	1.1 ± 0.4	-
	B	1.0 ± 0.4	-
	C	1.0 ± 0.3	-
	D	1.0 ± 0.3	-
	E	1.0 ± 0.4	-
	All	1.0 ± 0.4	-
Three beads	A	1.0 ± 0.3	-
	B	1.1 ± 0.5	-
	C	1.3 ± 0.5	-
	D	0.9 ± 0.3	-
	E	1.0 ± 0.3	-
	All	1.1 ± 0.4	-
